# Difficult Diagnosis in a Case of Pregnancy

**Published:** 1910-04-02

**Authors:** E. M. Magill


### DIFFICULT DIAGNOSIS IN A CASE OF PREGNANCY.

By E. M. MAGILL, L.S.A., M.C.P. and S., Alta.

The following are the notes of a case of pregnancy which gave considerable trouble in diagnosis on account of the absence of all definite signs of pregnancy in a patient who was a primipara at the age of 43, when more general disturbance might reasonably be looked for. From six weeks after conception to the completion of the fifth month the patient was under my constant supervision, and was repeatedly examined with the view of determining her pregnant, but only to get inconclusive results.

The past history was uneventful. Mrs. T. had been married five years when I first saw her. She had had no children, but gave a history of a questionable miscarriage at two months three years previously. She had always enjoyed excellent health, and was at the time of consultation a stout, healthy woman.

*March* 13, 1908.—She consulted me first for amenorrhœa; her last period had begun on January 22 and lasted five days. Before then she had been invariably regular, and was now afraid she was entering upon the change of life. She gave a history of palpitations, hot flushes, indigestion, constipation, nervousness, and pains in the back. She had had no suggestion of morning sickness, and did not think it possible that she could be pregnant. Per vaginum there was no blueing of the parts, the cervix was not softened, the uterus was in good position and not enlarged; the ovaries could not be felt.

*March* 28.—Cervix questionably softened and the uterus questionably globular, but owing to the thickness and resistance of the abdominal walls this was difficult to make out. No blueing of the parts, no tenseness of the breasts, no morning sickness or any other constitutional disturbance.

*April* 11.—Uterus enlarged to the size of an orange, but softening does not continue, and no other signs of pregnancy are manifested.

*April* 26.—Uterus continues to enlarge; no definite signs of pregnancy; general health continues good, though patient is anxious about her condition.

*May* 14.—A vague, indefinite tumour to be detected to within 1 inch of the umbilicus, very difficult to define owing to the large quantities of fat in the abdominal walls and the rigidity of the patient. No other signs of pregnancy.

*June* 20.—An examination under an anæsthetic was arranged for, as the patient's general health was beginning to fail rapidly, and she was becoming thin and very nervous. A very indefinite tumour could be made out reaching to the umbilicus and moving slightly with the cervix, which was now soft and patulous. The consistency of the tumour did not in the least suggest the pregnant uterus, being hard, unyielding, and tense, and its surface quite smooth. After repeated manipulations no contractions could be obtained and no fœtal movements detected. There was no suggestion of fœtal parts, the whole tumour appearing uniform and suggesting a fluid swelling only. Ballottement could not be obtained. The fœtal heart was continually listened for, but without success, both by myself and another medical practitioner. The patient has felt no suggestion of quickening, and there is no swelling or pigmentation of the breasts.

*June* 26.—Patient lost a quantity (about 2 pints) of clear fluid without colour or odour. On examination abdominally, the tumour was found to have decreased to three-quarters of its former size. Per vaginum the external os was very dilated, the internal os not dilated. The tumour was more distinct, but no uterine contractions occurred or fœtal movements, and the fœtal heart not heard. At 12 noon, however, uterine contractions set in strongly, and continued for 15 hours in spite of the free administration of opium. At 3 a.m., June 27, the pains still were very vigorous, and the patient was in a state of extreme exhaustion, but the internal os was not much affected. Believing that the flow of fluid above described to be the liquor amnii, and that the membranes were ruptured, and also that the fœtus was dead and abortion inevitable, the rigid internal os was dilated under chloroform. On intra-uterine examination the membranes were apparently intact, and on rupturing them a definite flow of liquor amnii was obtained. The fœtus when removed showed a full six months' development and gave a few signs of life.

The patient made an uneventful recovery, only marked by an excessive formation of milk, which gave some trouble. The difficulty of the case was the complete absence of all signs of pregnancy except amenorrhœa and a growing tumour, which in its tense and fluid consistency suggested much more a rapidly forming ovarian tumour than the pregnant uterus. During the last few weeks also the patient was losing flesh to a large extent, which made the possibility of malignancy come into one's mind. The fluid which came away after examination was apparently external to the amnion, had masked all signs of fœtal parts and movements, had deadened the heart sounds, and had apparently caused the perplexing consistency of the tumour. I do not know if an accumulation of fluid between chorion and amnion is at all frequent, but that is the solution suggested to me.

